# Differential diagnosis and prognosis of small renal masses: association with collateral vessels detected using contrast-enhanced computed tomography

**DOI:** 10.1186/s12885-022-09971-w

**Published:** 2022-08-05

**Authors:** Masato Yanagi, Tomonari Kiriyama, Jun Akatsuka, Yuki Endo, Hayato Takeda, Akifumi Katsu, Yuichiro Honda, Kyota Suzuki, Yoshihiro Nishikawa, Shunsuke Ikuma, Hikaru Mikami, Yuka Toyama, Go Kimura, Yukihiro Kondo

**Affiliations:** 1 Department of Urology, Nippon Medical School Hospital, 1-1-5, Sendagi, Bunkyo-ku, Tokyo, 113-8603 Japan; 2 Department of Radiology, Nippon Medical School Hospital, 1-1-5, Sendagi, Bunkyo-ku, Tokyo, 113-8603 Japan

**Keywords:** Collateral vessel, Small renal masses, Fat-poor angiomyolipoma, Renal cell carcinoma, Contrast-enhanced computed tomography, Diagnostic accuracy

## Abstract

**Background:**

Active surveillance (AS) is one of the treatment methods for patients with small renal masses (SRMs; < 4 cm), including renal cell carcinomas (RCCs). However, some small RCCs may exhibit aggressive neoplastic behaviors and metastasize. Little is known about imaging biomarkers capable of identifying potentially aggressive small RCCs. Contrast-enhanced computed tomography (CECT) often detects collateral vessels arising from neoplastic angiogenesis in RCCs. Therefore, this study aimed to evaluate the association between SRM differential diagnoses and prognoses, and the detection of collateral vessels using CECT.

**Methods:**

A total of 130 consecutive patients with pathologically confirmed non-metastatic SRMs (fat-poor angiomyolipomas [fpAMLs; *n* = 7] and RCCs [*n* = 123]) were retrospectively enrolled. Between 2011 and 2019, SRM diagnoses in these patients were confirmed after biopsy or surgical resection. All RCCs were surgically resected. Regardless of diameter, a collateral vessel (CV) was defined as any blood vessel connecting the tumor from around the kidney using CECT. First, we analyzed the role of CV-detection in differentiating between fpAML and RCC. Then, we evaluated the sensitivity, specificity, positive predictive value (PPV), negative predictive value (NPV), and accuracy of RCC diagnosis based on CV-detection using CECT. We also assessed the prognostic value of CV-detection using the Fisher exact test, and Kaplan-Meier method and the log-rank test.

**Results:**

The sensitivity, specificity, PPV, NPV, and accuracy of CV-detection for the diagnosis of small RCCs was 48.5, 45.5, 100, 100, and 9.5% respectively. Five of 123 (4.1%) patients with RCC experienced recurrence. CV-detection using CECT was the only significant factor associated with recurrence (*p* = 0.0177). Recurrence-free survival (RFS) was significantly lower in patients with CV compared with in those without CV (5-year RFS 92.4% versus 100%, respectively; *p* = 0.005). In addition, critical review of the CT images revealed the CVs to be continuous with the venous vessels around the kidney.

**Conclusions:**

The detection of CVs using CECT is useful for differentiating between small fpAMLs and RCCs. CV-detection may also be applied as a predictive parameter for small RCCs prone to recurrence after surgical resection. Moreover, AS could be suitable for small RCCs without CVs.

**Supplementary Information:**

The online version contains supplementary material available at 10.1186/s12885-022-09971-w.

## Background

The frequency of detection of small renal masses (SRMs; < 4 cm) has increased owing to the recent advancements in imaging modalities and their widespread use. For SRMs, differentiating between benign and malignant tumors based on imaging findings can be challenging. Fat-poor angiomyolipoma (fpAML) is a benign tumor that is difficult to distinguish from renal cell carcinoma (RCC). Some fpAMLs are pathologically diagnosed as benign tumors after surgical resection [1–3]. Oncocytoma is another common benign tumor occurring predominantly in individuals of European descent and with a relatively low incidence in Asian populations [4]. However, the incidence of fpAML is equivalent in Asian and Western populations [4, 5], and the preoperative differentiation of fpAML from RCC is broadly relevant. Although percutaneous biopsy is often effective in differentiating between fpAMLs and RCCs [6], there is a need to improve the accuracy of noninvasive imaging approaches to screening.

Computed tomography (CT), which has been widely used for the evaluation of renal masses, is less expensive than magnetic resonance imaging [7]. Several studies have reported that quantitative analyses of CT data are helpful for the diagnosis of fpAMLs [8–11]. However, some imaging findings are common to both fpAML and RCC. Regarding contrast-enhanced computed tomography (CECT), imaging methods vary among institutions. To facilitate the accurate diagnosis of SRMs, useful, accessible, and well-defined CT features that are detectable independently from applied contrast media and imaging protocol are needed.

The morphological features of SRMs are consistent, regardless of the CT imaging protocol. Morphological CT findings, such as the overflowing beer sign (OBS) and angular interface (AI), are valuable predictors of fpAML and help differentiate fpAMLs from RCCs [11–13]. OBS is a more accurate imaging predictive biomarker of small (≦4 cm) fpAML than AI [13]. Previously, collateral or perirenal blood vessels were detected in some RCCs using CECT, regardless of CECT protocol. However, these features were not observed in any fpAMLs [11, 14]. Accordingly, we hypothesized that collateral vessels (CVs) may be a distinguishing characteristic of RCCs on CECT images.

In general, small RCCs do not metastasize and are often treated during active surveillance (AS) to avoid surgical intervention [15, 16]. However, a small number of small RCCs may exhibit aggressive behavior and metastasize [15, 16]. Interventions for the complete cure of metastatic RCC are generally challenging and may increase the risk of mortality. Therefore, there is a need for screening methods that can detect potentially aggressive small RCCs among SRMs.

Some recurrent small RCCs after surgical resection presented with CVs on preoperative CECT images, independently from the timing of contrast enhancement. The purpose of this study was to investigate whether the presence of CVs on CECT images could be useful for differentiating between small fpAMLs and RCCs, and whether CV-detection is a factor associated with recurrence after surgical resection of small RCCs.

## Methods

### Patient population

We retrospectively reviewed our pathological database and retrieved the data of 165 consecutive patients with 168 pathologically-documented non-metastatic SRMs (seven fpAMLs, 161 RCCs, and zero oncocytomas) treated at Nippon Medical School Hospital between January 2011 and December 2019. Patient characteristics, including sex, age, body mass index (BMI), Eastern Cooperative Oncology Group performance status (ECOG PS), Charlson Comorbidity Index, tumor size, tumor laterality, tumor location, CT findings, treatment methods, pathological information, and clinical outcomes were evaluated.

**Fig. 1:**
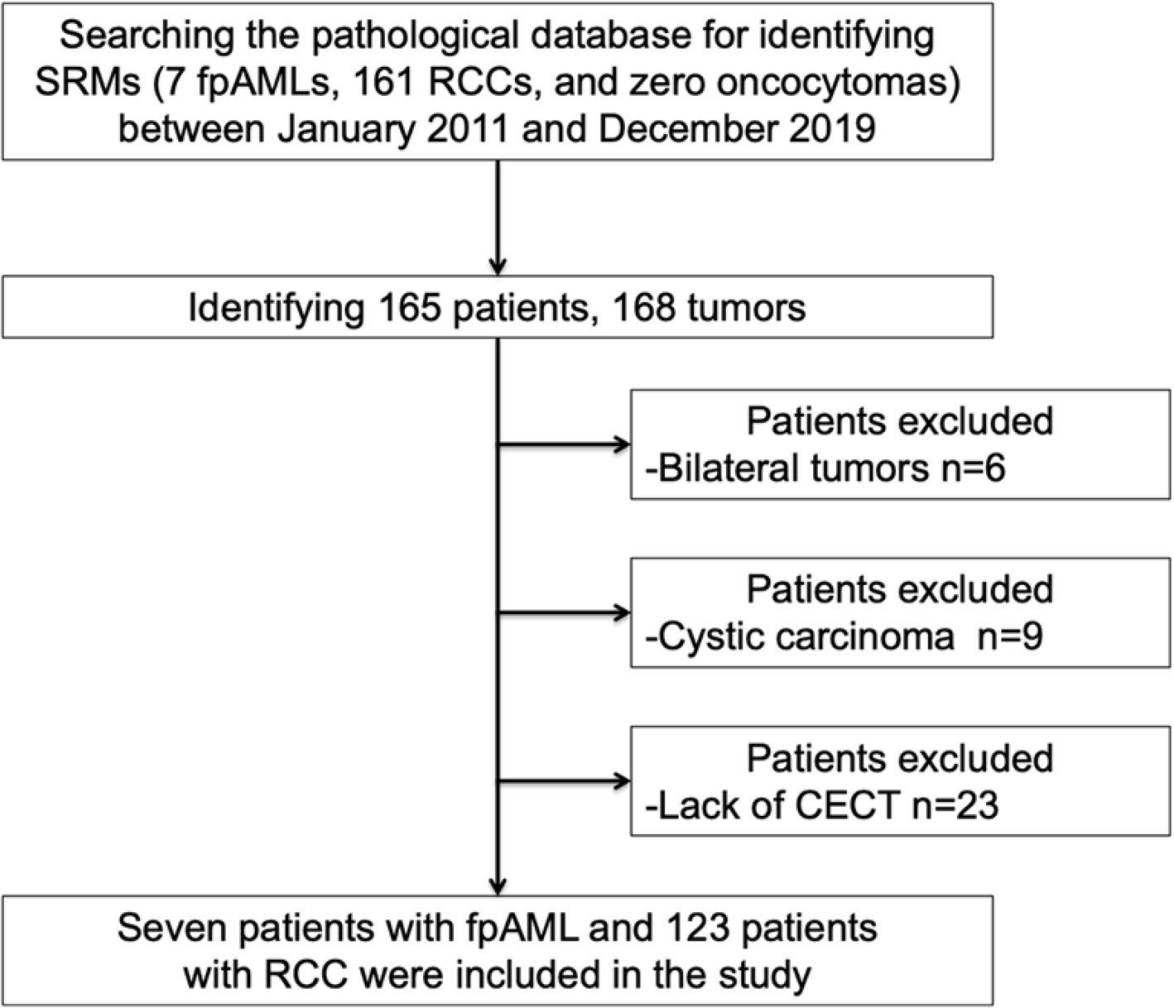
Flowchart of the patient selection procedure

All included SRMs measured less than 4 cm and lacked an apparent fat component on unenhanced CT. Pathological diagnoses were determined after biopsy or surgical resection. Patients with bilateral (*n* = 3; six tumors) or cystic tumors (*n* = 9) as well as patients who did not undergo CECT (*n* = 23) were excluded. Finally, 130 SRMs, which included seven fpAMLs and 123 RCCs (105 clear cell RCCs, eight papillary RCCs, seven chromophobe RCCs, one mucinous tubular and spindle cell carcinoma, and two unclassified RCCs) were included in this study (Fig. 1). At our institution, we generally only perform renal biopsy for patients with suspected benign masses who are willing to undergo partial or radical nephrectomy if the biopsy reveals the mass to be malignant. All RCCs were treated with surgical resection, and treated patients were regularly followed-up using blood tests and CT scan after surgical resection.

This study was performed in line with the principles of the Declaration of Helsinki. The study design was approved by the Ethics Committee at Nippon Medical School Hospital (approval number 29-11-861). Based on the retrospective nature of the study, the need for written informed consent was waived by the Ethics Committee at Nippon Medical School Hospital. However, all participants had the opportunity to opt-out on a homepage of the Ethics Committee at Nippon Medical School Hospital.

### Imaging evaluations

**Fig. 2:**
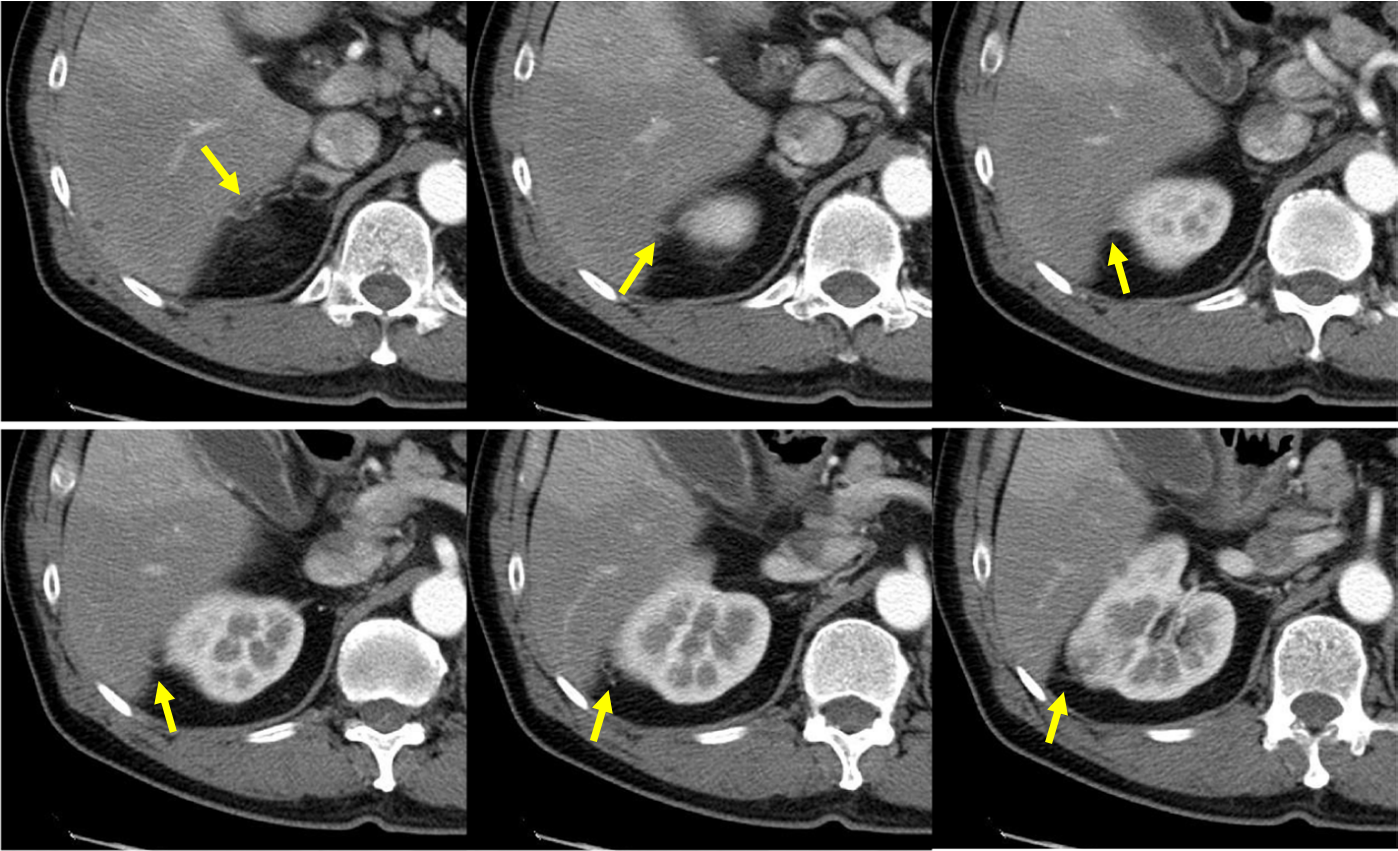
Representative images showing the appearance of CVs on CECT. We defined CVs as blood vessels of any diameter, with a definite connection between the tumor and the perirenal region. The yellow arrows indicate the CVs. CV, collateral vessel; CECT, contrast-enhanced computed tomography

**Fig. 3:**
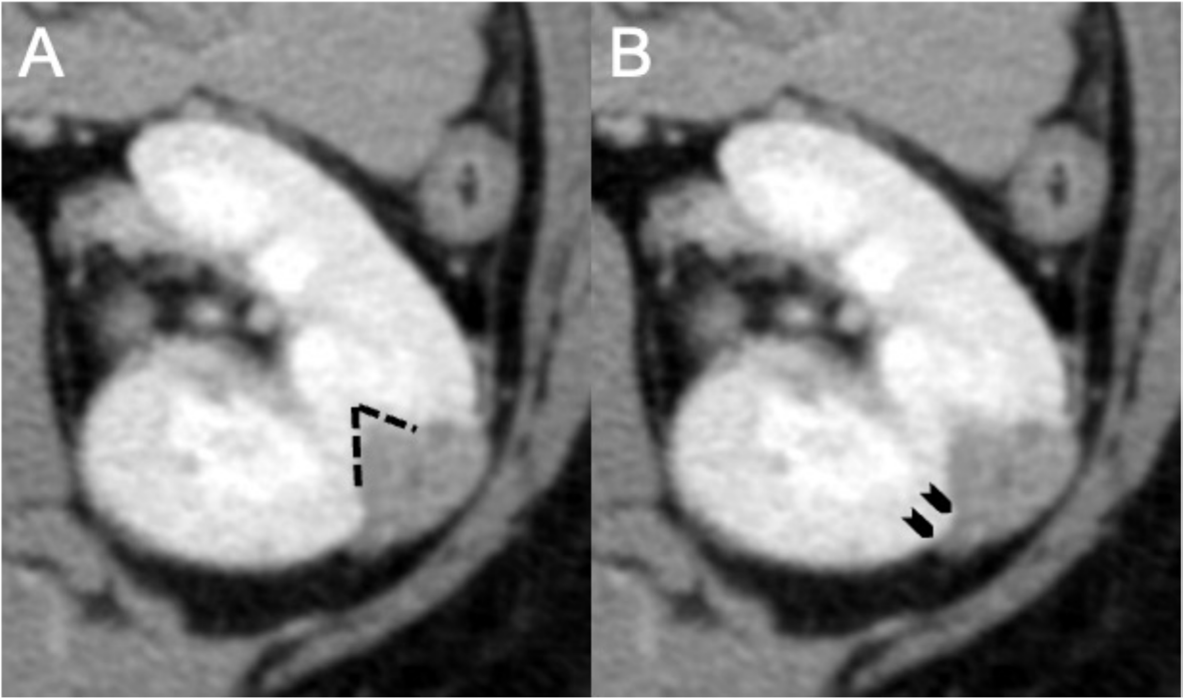
Representative images showing the appearance of AI and OBS on CECT. **A** The AI in the parenchymal phase was defined as an angle of 90° or less (black lines). (B) A contact length ≥ 3 mm of the bulging portion of the renal tumor onto the renal surface was indicative of a positive OBS (black arrows). AI, angular interface; OBS, overflowing beer sign; CECT, contrast-enhanced computed tomography

A urologist with 12 years of experience collected the CECT data. Subsequently, a urologist and radiologist with 13 and 19 years of experience in genitourinary imaging, respectively, independently analyzed the CECT images. Both assessors were blinded to the patients’ pathological information and clinical outcomes, and any diagnostic discrepancies were resolved by consensus. In the present study, abdominal CT images of 5-mm slice thickness were obtained using various 64- to 320-channel multi-detector scanners, both at the hospital and at other facilities. There was no uniform scan protocol; however, an example of a typical scan protocol is shown below. Non-contrast enhanced acquisition of the entire abdomen and pelvis was followed by triphasic dynamic CT, if available. The scan durations were corticomedullary phase, 30-40 sec after the injection; nephrographic phase, approximately 90–100 sec; and excretory delayed phase, approximately 300 sec. The scanning parameters were tube voltage, 120 keV; auto mA modulation; field of view, 300-400 mm; gantry rotation time, 0.4-0.5; collimation, 40 mm^− 80^ mm; pitch, 0.9-1.0. Regardless of vessel diameter, any blood vessel around the kidney with a confirmed connection to the tumor identified using CECT was defined as a CV (Fig. 2). While CVs can be detected on unenhanced CT, they can often be incorrectly diagnosed as reno-renal fascial septa, reno-fascial septa, and perirenal fat stranding. However, these features are not enhanced on CECT. Therefore, we confirmed CV on images taken from the corticomedullary and nephrographic phases of CECT. The AI and OBS were also analyzed using CECT during the parenchymal phase based on the algorithm proposed by Kim et al. (Fig. 3A and B) [13].

The location, shape (round or non-round), and CT value (HU: Hounsfield Unit) of the tumor on unenhanced CT as well as the enhancement pattern (homogeneous or heterogeneous) in the corticomedullary phase were reviewed. To measure the CT value, the region-of-interest was set as the largest area of the tumor on the axial image.

### Statistical analysis

Statistical analyses were performed using JMP® version 13 (SAS Institute Inc., Cary, NC, USA). *P* < 0.05 was considered statistically significant. Categorical variables were compared using the Fisher’s exact test. Continuous variables were compared using either the t-test or Mann-Whitney U test, depending on the results of the one-sample Kolmogorov–Smirnov test. The sensitivity, specificity, positive predictive value (PPV), negative predictive value (NPV), and accuracy of CV-detection applied to small RCC diagnosis using CECT were evaluated. The sensitivity, specificity, PPV, NPV, and accuracy of AI and OBS applied to the detection of small fpAMLs using CECT were also evaluated. Survival curves were generated using the Kaplan–Meier method, and between-group differences were evaluated using the log-rank test.

## Results

### Patient characteristics

Table 1 summarizes the characteristics of 130 patients with SRMs included in this study (i.e., one SRM per patient). The proportion of women was significantly higher in the fpAML group than in the RCC group (fpAML, 71.4%; RCC, 18.7%; *p* = 0.0052). BMI was significantly lower in the fpAML group than in the RCC group (*p* = 0.0284).

**Table 1:** Clinical characteristics of patients in the study population (*n* = 130)

Variables	fpAML (n = 7)	RCC (n = 123)	*P*-value
Sex (Female); n (%)	5 (71.4)	23 (18.7)	*0.0052
Age (years); median (IQR)	50 (38–64)	64 (53–70)	0.0726
BMI (kg/m^2^); median (IQR)	21.1 (20.0–23.5)	24.1 (21.2–26.8)	*0.0284
ECOG PS (0/≥1)	7/0	116/7	1.0000
Charlson comorbidity index (0/≥1)	7/0	99/24	0.3477

*fpAML* fat-poor angiomyolipomas, *RCC* Renal cell carcinoma, *IQR* Interquartile range, *BMI* Body mass index, *ECOG PS* Eastern Cooperative Oncology Group Performance Status. *p < 0.05

### Imaging differentiation between fpAML and RCC

The mean CT value of fpAMLs was higher than that of RCCs (*p* = 0.0339). The homogeneity of tumor enhancement (*p* = < 0.0001) was significantly associated with fpAML diagnoses. Positive OBS (*p* < 0.0001) and AI (*p* = 0.0018) were significantly associated with fpAML detection. On the other hand, the presence of CVs (*p* = 0.0192) was significantly associated with RCCs (Table 2). CVs could not be detected in AML (Supplementary Fig. 1). The diagnostic performance of using either OBS or AI for fpAML diagnosis as well as using CV-detection for RCC diagnosis is summarized in Table 3. All CVs from RCCs were continuous with the renal capsule vein or other venous vessels.

**Table 2:** Radiological characteristics of SRMs on CT

Variables	fpAML (n = 7)	RCC (n = 123)	P-value
Tumor size (mm); median (IQR)	25 (22–32)	26 (20–33)	0.8081
Tumor size (≤2 cm vs. > 2 cm)	1/6	26/97	1.0000
Tumor location			
Right/Left	2/5	54/69	0.6981
Posterior/anterior	5/2	74/49	0.1263
Lateral/Medial	4/3	76/47	1.0000
Exophytic/Endophytic	7/0	116/7	1.0000
Tumor shape (Round/Non-round)	2/5	68/55	0.4660
Tumor attenuation in the unenhanced phase (HU); median (IQR)	43 (26 − 49)	31 (25 − 38)	*0.0339
Homogeneity/Heterogeneity	6/1	16/107	* < 0.0001
OBS (+/−)	3/4	0/123	* < 0.0001
AI (+/−)	4/3	9/114	*0.0018
CV (+/−)	0/7	56/67	*0.0192

CT, computed tomography; fpAML, fat-poor angiomyolipomas; RCC, renal cell carcinoma; IQR, interquartile range; HU, Hounsfield Unit; OBS, overflowing beer sign; AI, angular interface; CV, collateral vessel. *p < 0.05

**Table 3:** Diagnostic performance of OBS and AI for fpAML, and CV-detection for RCC

	Sensitivity (%)	Specificity (%)	PPV (%)	NPV (%)	Accuracy (%)
OBS	42.9	100	100	96.9	96.9
AI	57.1	92.7	30.8	97.4	90.8
CV	45.5	100	100	9.5	48.5

*PPV* Positive predictive value, *NPV* Negative predictive value, *OBS* Overflowing beer sign, *AI* Angular interface, *CV* Collateral vessel

### Prognostic factors for small RCCs

**Fig. 4:**
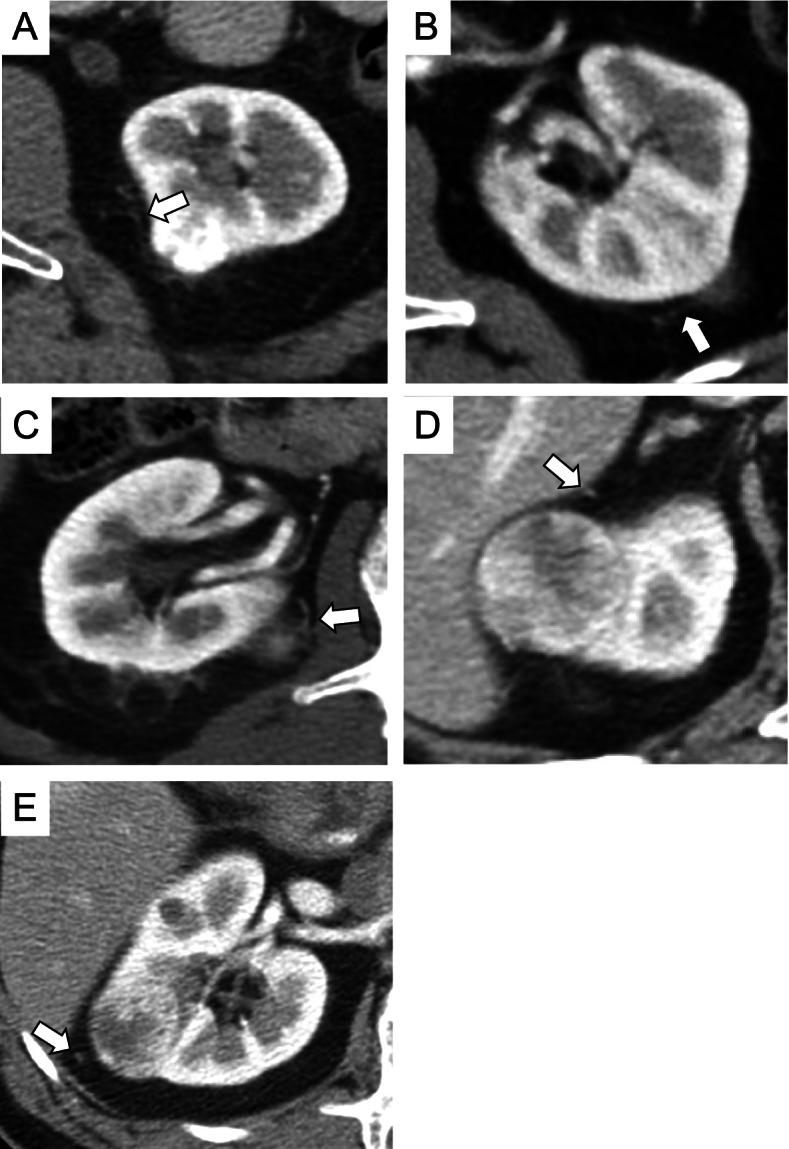
CVs in five patients with RCC and recurrence. **A**–**E** The white arrows indicate CVs detected in the five patients with RCC and recurrence. CV, collateral vessel; RCC, renal cell carcinoma

**Fig. 5:**
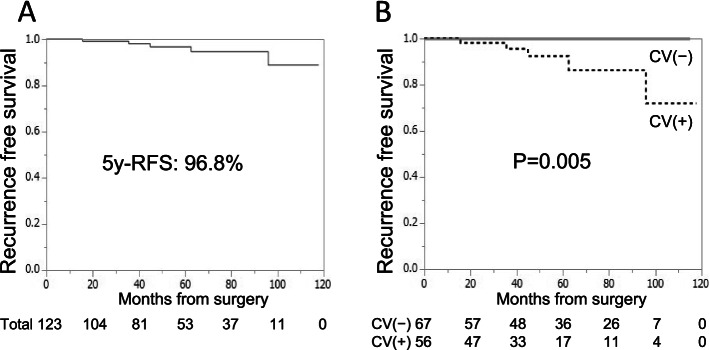
Kaplan–Meier curves depicting the 5-year RFS in patients with small RCC after surgical resection. **A** The 5-year RFS after surgical resection of all patients with small RCCs was 96.8%. **B** Patients with CV had a significantly worse 5-year RFS than those with CV (*p* = 0.005). The 5-year RFS of patients with and without CV was 92.5 and 100%, respectively. RFS, recurrence-free survival; RCC, renal cell carcinoma; CV, collateral vessel

All patients with RCC were treated with nephrectomy or partial nephrectomy. The median duration of post-surgery follow-up of patients with RCC was 54.6 months. The clinicopathological factors of patients with and without recurrence are shown in Table 4. Five (4.1%) of the 123 patients with RCC experienced tumor recurrence. In these five patients, the surgical margins were negative, but CVs were present in all 5 cases (Fig. 4). Three of the five patients had recurrence with lung metastases. One patient had ipsilateral renal recurrence, lung metastasis, and lymph node metastasis. The fifth patient had an ipsilateral retroperitoneal metastasis. The pathological factors common to all five patients with recurrence were clear cell carcinoma (CCC), not Fuhrman grade ≥ 3, infiltrative growth (INF) ≥ β, central necrosis (+), and microvascular invasion (MVI) (+) (Table 4). The 5-year recurrence-free survival (RFS) for all patients with resected RCC was 96.8% (Fig. 5A). The presence of CVs was the only significant predictor of recurrence after surgical resection (*p* = 0.0177) (Table 4). The 5-year RFS was significantly worse in patients with CV than in those without CV (92.5% versus 100%; *p* = 0.005) (Fig. 5B).

**Table 4:** Clinicopathological characteristics of patients with RCC with and without recurrence

Variables	With recurrence (*n* = 5)	Without recurrence (*n* = 118)	P-value
Sex (Male/Female)	5/0	95/23	0.3484
Age (years); median (IQR)	65 (60–72)	64 (53–70)	0.5642
Age (≦60 vs 60 < years)	1/4	50/68	0.4017
BMI (kg/m^2^); median (IQR)	24.5 (21.5–28.3)	24.1 (21.2–26.7)	0.8177
ECOG PS (0/≥1)	5/0	111/7	1.0000
Charlson comorbidity index (0/≥1)	5/0	94/24	1.0000
Tumor size (mm); median (IQR)	30 (20–36)	26 (20–33)	0.5766
Tumor size (≦ 2 cm vs > 2 cm)	1/4	25/93	1.0000
Tumor location			
Right/Left	3/2	51/67	0.3849
Posterior/anterior	4/1	45/73	0.0814
Lateral/Medial	3/2	73/45	0.7163
Exophytic/Endophytic	5/0	111/7	1.0000
Tumor shape (Round/Non-round)	1/4	54/64	0.3790
Homogeneity/ Heterogeneity	0/5	26/92	0.2983
CV (+/−)	5/0	51/67	*0.0177
Surgical method (Partial/Radical)	5/0	103/15	0.5159
Histological type (CCC/non-CCC)	5/0	100/18	0.4469
pT stage (1a/ 3a)	4/1	105/13	0.4593
Fuhrman grade (1,2/ 3,4)	3/2	95/23	0.2678
INF (α/β,γ)	2/3	88/30	0.1193
Central necrosis (+/−)	1/4	25/93	0.7017
MVI (+/−)	3/2	50/68	0.3700
Surgical margin (+/−)	0/5	3/115	1.0000

*IQR* Interquartile range, *BMI* Body mass index, *ECOG PS* Eastern Cooperative Oncology Group Performance Status, *CV* Collateral vessel, *CCC* Clear cell carcinoma, *INF* Infiltrative growth, *MVI* microvascular invasion *p < 0.05

### Characteristics of patients with CVs from RCCs

Table 5 summarizes the comparative clinicopathological characteristics of patients with and without CVs from small RCCs. Patients with CVs were older (*p* = 0.0012) and had a worse ECOG PS (*p* = 0.0033) than those without CVs. The frequency of CV-detection in cases of a CCC histological type was significantly higher than that in non-CCC cases (*p* = 0.0405). The enhancement pattern in the corticomedullary phase and tumor shape differed significantly between small RCCs with and without CVs (*p* < 0.0001). RCCs with CVs were significantly larger (p < 0.0001) and had worse pathological findings than RCCs without CVs (pT3a: *p* = 0.0482; MVI (+): *p* = 0.0172).

**Table 5:** Clinicopathological characteristics of RCC patients with and without CVs

Variables	CV (+) *n* = 56	CV (−) *n* = 67	P-value
Sex (male); n (%)	47 (83.9)	53 (79.1)	0.6432
Age (years); median (IQR)	67 (59–72)	59 (49–68)	*0.0012
BMI (kg/m^2^); median (IQR)	23.5 (20.8–26.5)	24.9 (21.3–27.2)	0.1470
ECOG PS (≥1); n (%)	7 (12.5)	0 (0)	*0.0033
Charlson comorbidity index (≥1); n (%)	22 (39.3)	11 (16.4)	0.3693
Tumor size (mm); median (IQR)	30 (25–35)	23 (17–30)	* < 0.0001
Tumor size > 2 cm	51 (91.1)	46 (68.7)	*0.0034
Tumor location; n (%)			
Right	25 (44.6)	29 (43.3)	1.0000
Posterior	23 (41.1)	26 (38.8)	0.8543
Lateral	38 (67.9)	38 (56.7)	0.2639
Exophytic; n (%)	55 (98.2)	61 (91.0)	0.1248
No round; n (%)	43 (76.8)	25 (37.3)	* < 0.0001
Heterogeneity; n (%)	50 (89.3)	47 (70.1)	*0.0058
CCC; n (%)	52 (92.9)	53 (79.1)	*0.0405
Papillary RCC; n (%)	3 (5.3)	5 (7.5)	0.7266
Chromophobe RCC; n (%)	1 (1.8)	6 (9.0)	0.1248
Other types of RCC; n (%)	0 (0.0)	3 (4.4)	0.2499
pT3a; n (%)	10 (17.8)	4 (6.0)	*0.0482
Fuhrman grade 3, 4; n (%)	14 (25.0)	11 (16.4)	0.2669
INFβ, γ; n (%)	20 (35.7)	13 (19.4)	0.0650
Central necrosis (+); n (%)	12 (21.4)	14 (20.9)	1.0000
MVI (+); n (%)	31 (55.4)	22 (32.8)	*0.0172

*CV* Collateral vessel, *IQR* Interquartile range, *BMI* Body mass index, *ECOG PS* Eastern Cooperative Oncology Group Performance Status, *CCC* Clear cell carcinoma, *RCC* Renal cell carcinoma, *INF* Infiltrative growth, *MVI* Microvascular invasion *p < 0.05

## Discussion

Studies on organs such as the thyroid gland and uterus have shown that the evaluation of vascular patterns allows for accurate differential diagnosis using ultrasonography [17, 18]. Previous studies on renal tumors revealed that evaluating the vascular pattern using CECT permits an accurate differential diagnosis between fpAML and RCC [11, 14]. In the study by Bagheri et al. [14], peritumor vessels were defined as those with a diameter of 2 mm or greater. In the present study, the diameter of the peritumoral vessels was very small because only SRMs were analyzed. Therefore, we defined CVs as blood vessels of any diameter that were confirmed to be continuous with the tumor from the perirenal region.

According to previous studies, 21.8 to 24.5% of RCCs, including non-small RCCs, had perirenal vessels or CVs [11, 14]. In our study, CVs were observed in 45.5% (56/123) of RCCs using CECT, but none were observed in fpAMLs. The specificity and PPV of CV-detection for identifying RCCs were both 100%. These results suggest that CVs are characteristic of small RCCs and may be useful in the differential diagnosis of SRMs. The results of morphologic analyses related to fpAMLs in this study and previous studies were similar [13], suggesting that OBS and AI are characteristic morphological findings of small fpAML and are also useful for the diagnosis of SRMs. OBS is a new morphological feature of SRMs proposed by Kim et al. [13]. According to our findings, the specificity and PPV of AI were lower than those of OBS (92.7% versus 100 and 30.8% versus 100%, respectively). Compared with using AI, these findings suggest that patients with CVs or a positive OBS are more likely to be correctly diagnosed as having RCCs or fpAMLs, respectively.

In the present study, the presence of CVs on CECT images was the only significant predictor of recurrence of small RCCs after surgical resection. In contrast, previous studies identified the following risk factors for recurrence after surgical resection of SRMs: large tumor size at presentation [16], age greater than 60 years at diagnosis [19], high Fuhrman grade, and presence of lymphovascular invasion [20]. Our finding suggests that CVs on CECT images may also be useful in identifying small RCCs more likely to recur after surgical resection. In this study, factors associated with CV-detection included older age, a poor ECOG PS, large tumor size, non-round shape, heterogeneity of tumor enhancement, and pathological features related to poor clinical outcomes [pT3a and MVI (+)]. Therefore, it is reasonable to infer that small RCCs with CVs have a worse prognosis than those without CVs.

Among the three major pathological subtypes of RCC, namely CCC, papillary RCC, and chromophobe RCC [21], CCC is most common and is suggestive of a hypervascular tumor [22]. In the present study, the frequency of CVs in CCC was significantly higher than in non-CCC (Table 5), supporting the premise of a higher angiogenic capacity in CCC. In addition, detailed review of the CT images showed that CVs were continuous with venous vessels surrounding the kidney, suggesting that microscopic metastases entering the venous circulation from the tumor may be draining into the systemic venous circulation and adjacent organs. This may explain the association between CVs and an increased risk of recurrence after surgical resection.

Detecting aggressive RCCs at initial diagnosis is important for planning treatment strategies. CV may be useful for determining which cases require surgical resection and more stringent subsequent follow-up. Furthermore, CECT is widely applied when diagnosing SRMs and may be easy to use for the evaluation of CVs because their detection does not depend on the acquisition timing.

This study had several limitations. First, the study design was retrospective and utilized a small patient cohort from a single institution, which precluded multivariate analysis. Second, the median post-surgery follow-up period for patients with RCC was 54.6 months. Small RCCs may recur 10 years after surgical resection [23]; therefore, the follow-up period may not have been long enough to identify all cases of recurrence. Considering this, a prospective, multicenter study with a larger sample size and a longer follow-up period is warranted. Third, since we used data from a pathology database, the cohort of this study did not include patients with SRMs, without a pathological diagnosis, that had been managed with active surveillance and surgical selection criteria are not specified. Therefore, surgical selection bias might be present. However, we generally only perform renal biopsy for patients who can undergo partial or radical nephrectomy if the biopsy reveals a malignant mass. Therefore, we believed that this limitation did not have a major impact on the results of this study. Fourth, a uniform CT imaging and contrast protocol was not implemented throughout the study. However, given that all SRMs in our cohort were diagnosed by CECT, including the unenhanced, corticomedullary, nephrographic and excretory phases, at 5-mm slice thickness, this issue should not significantly impact our imaging analyses. Fifth, since this was a retrospective study, CECT was not performed for the purpose of detecting CVs. Unfortunately, since no additional raw data is available, we are unable to reconstruct images at less than 5-mm slice thickness. For more accurate detection of CVs, further studies using CECT with less than 5-mm slice thickness are required. Sixth, we did not detect CVs in completely endophytic masses. While assessment of CVs might not be useful for completely endophytic masses, given the very small sample in this study, further studies with large cohorts are required to draw definitive conclusions. In addition, in our study, the RCCs with CVs were significantly larger than those without CVs. Therefore, there might be confounding between tumor size and presence of CVs. Further studies with larger cohorts and multivariate analyses are required. Finally, in the present study, patients were treated with surgical resection before metastases. Therefore, we could not confirm whether metastases would appear in the absence of surgical resection. To overcome this limitation, we believe that a prospective study of non-resected small RCCs treated with AS is needed.

## Conclusions

The presence of CVs on CECT images may be useful to differentiate between small fpAMLs and RCCs. CV-detection may also be applied as a predictive parameter for small RCCs that are more prone to recurrence after surgical resection. Moreover, AS could be suitable for small RCCs without CVs.

## Supplementary Information

**Additional file 1.**

## Data Availability

The datasets used and/or analyzed during the current study are available from the corresponding author upon reasonable request.

## Abbreviations

AI: Angular interface
AS: Active surveillance
BMI: Body mass index
CCC: Clear cell carcinoma
CECT: Contrast-enhanced computed tomography
CT: Computed tomography
CV: Collateral vessel
ECOG PS: Eastern Cooperative Oncology Group Performance Status
fpAML: Fat-poor angiomyolipoma
HU: Hounsfield Unit
INF: Infiltrative growth
IQR: Interquartile range
NPV: Negative predictive value
MVI: Microvascular invasion
OBS: Overflowing beer sign
PPV: Positive predictive value
RCC: Renal cell carcinoma
RFS: Recurrence-free survival
SRM: Small renal mass
