# Supplementary material for: Differential diagnosis and prognosis of small renal masses: association with collateral vessels detected using contrast-enhanced computed tomography
Source: BMC Cancer. 2022 Aug 5;22:856. doi: 10.1186/s12885-022-09971-w (PMC9354334; doi:10.1186/s12885-022-09971-w)
Supplement: Supplementary file 1 — Additional file 1. [file 12885_2022_9971_MOESM1_ESM.pdf]

Supplementary Fig. 1 Representative images of AML showing the absence of CV on CECT

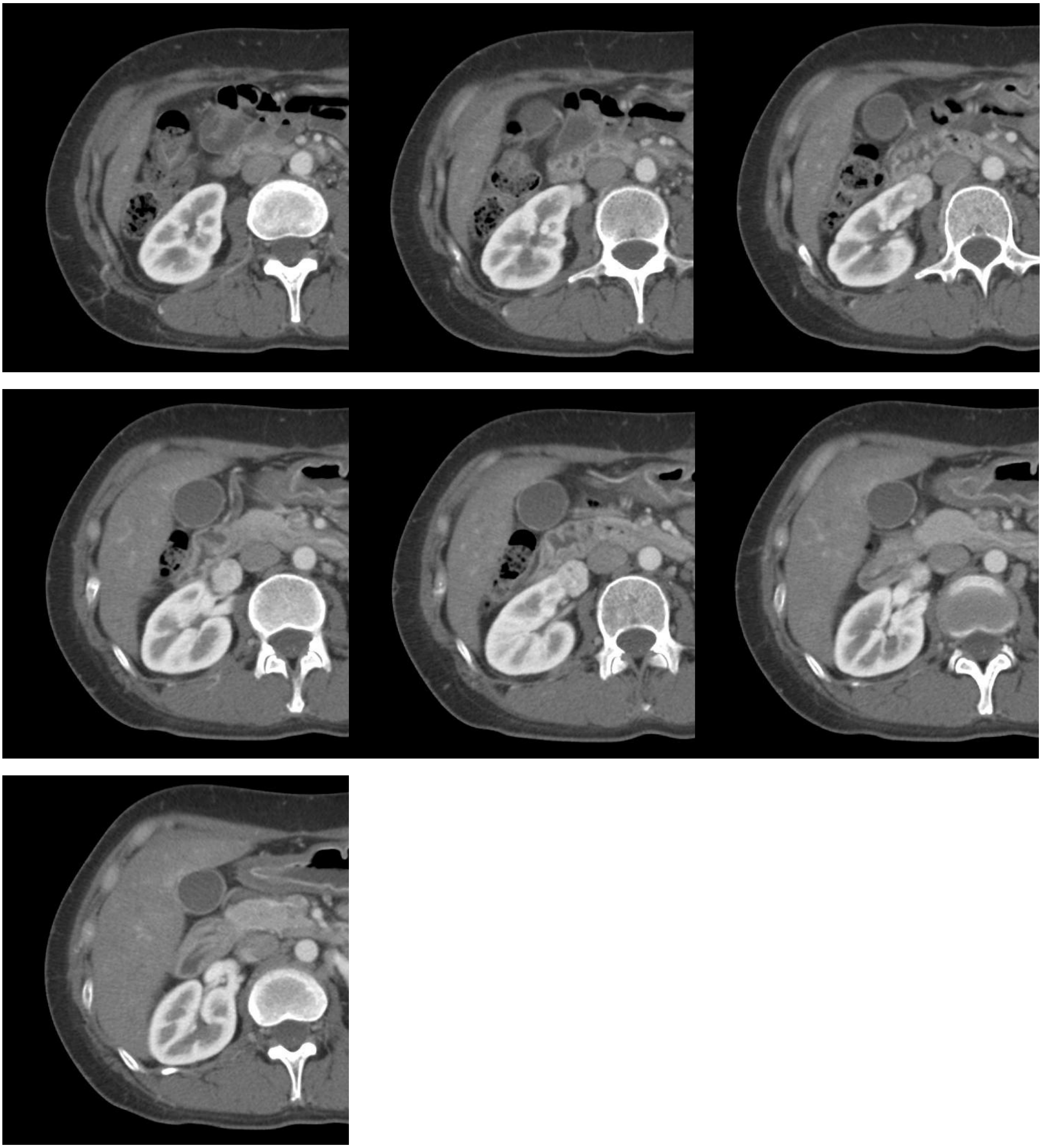

CVs could not be detected in AML.
AML, angiomyolipoma; CV, collateral vessel; CECT, contrast-enhanced computed tomography
